# Anti-Toothache

**Published:** 1879-09

**Authors:** 


Anti-toothache —Mr. James Mersou, L. D. S., writes to the *Brit. Jour. Dental Science*, that acute pain can often be suppressed by pungent aromatics, just as we know essential oils are popular remedies for toothache, as are creosote, peppers, spirits, etc. But, better still, he tells us that, combined with chloroform and aconite, they will prevent the pain of tooth extraction. Hundreds of patients told him they did not feel the pain. Here is his formula for a local anæsthetic to supersede chloroform, ether, the gas. etc.:

R. Chloroform, pur............3 drachms.

Tr. aconiti (Fleming's)......3 drachms.

Tr. capsici.................1 drachm.

Tr. pyrethri................½ drachm.

Ol. caryoph.................½ drachm.

Pulv. Camph.................½ drachm.

Mix.

The tooth and surrounding gums are to be previously dried, and then four or five drops of this applied with cotton wool. Then without delay use the forceps, but the instrument must be warmed. This is most important. We have felt the pang of the cold steel, and whether the anæsthetic or not be used, agree with the propriety of using warm instruments. For toothache, a pellet of cotton wool soaked in the above, may be introduced into the cavity, and is said often to give speedy relief.—*The Doctor.*

A Doctor who had one day allowed himself to drink too much, was sent for to see a fashionable lady who was ailing. He sat down by the bedside, took out his watch and began to count her pulse as well as his obfusticated condition would permit. He counted: "One, two, three," then he got confused, and began again: "One, two." No; he could not do it. Thoroughly ashamed of himself, he shut up his watch, muttering: "Tipsy, I declare—tipsy!" Staggering to his feet, he told the lady to keep her bed and take some hot lemonade, to throw her into a perspiration, and he would see her the next day. In the morning he received the following note from the lady, marked "private:"

"Dear Doctor: You were right. I dare not deny it. But I am thoroughly ashamed of myself, and will be more careful for the future. Please accept the enclosed fee for your visit (a ten pound note), and do not, I entreat of you, breathe a word about the state in which you found me."

The lady, in fact, had herself been drinking too much, and catching the doctor's murmured words, thought they referred to her. He was too far gone to see what was the matter with his patient, and she too far to observe that the doctor was in the same condition!—*Ex.*

---

It has just leaked out that the day Dr. Schliemann excavated the tomb of Agamemnon, just as he struck the monarch's sepulchre he heard a low chuckling laugh behind him, and a voice said, in good United States, "Here, quick! put him in here!" The doctor turned, and saw an Ohio Medical College student holding a big India rubber bag.—*Phil. Rev. of Medicine and Pharmacy.*

---

Treatment of Urticaria by Atropine.—In 1876 Dr. Fraenkel recommended the internal administration of atropine in the treatment of severe forms of urticaria. Dr. Schwimmer now reports three cases of this affection, which, after proving rebellious to all the usual methods of treatment, were rapidly cured by atropine. These three cases, however, show that the atropine treatment does not prevent relapse. Dr. Schwimmer believes that urticaria is simply an angioneurosis, and ascribes the efficacy of the drug to its action on the sympathetic system. —*Lyon Medical.*

